# N-acetylcysteine attenuates lipopolysaccharide-induced impairment in lamination of Ctip2-and Tbr1- expressing cortical neurons in the developing rat fetal brain

**DOI:** 10.1038/srep32373

**Published:** 2016-08-31

**Authors:** Ming-Wei Chao, Chie-Pein Chen, Yu-Hsiu Yang, Yu-Chen Chuang, Tzu-Yun Chu, Chia-Yi Tseng

**Affiliations:** 1 Department of Bioscience Technology, Chung Yuan Christian University, Zhongli district, Taoyuan City, Taiwan; 2 Division of High Risk Pregnancy, Mackay Memorial Hospital, Taipei, Taiwan; 3 Department of Biomedical Engineering, Chung Yuan Christian University, Zhongli district, Taoyuan City, Taiwan; 4 International Master Program of Biomedical Material and Technology, Chung Yuan Christian University, Zhongli district, Taoyuan City, Taiwan; 5 Center for Nano-Technology, Chung Yuan Christian University, Zhongli district, Taoyuan City, Taiwan

## Abstract

Oxidative stress and inflammatory insults are the major instigating events of bacterial intrauterine infection that lead to fetal brain injury. The purpose of this study is to investigate the remedial effects of N-acetyl-cysteine (NAC) for inflammation-caused deficits in brain development. We found that lipopolysaccharide (LPS) induced reactive oxygen species (ROS) production by RAW264.7 cells. Macrophage-conditioned medium caused noticeable cortical cell damage, specifically in cortical neurons. LPS at 25 μg/kg caused more than 75% fetal loss in rats. An increase in fetal cortical thickness was noted in the LPS-treated group. In the enlarged fetal cortex, laminar positioning of the early born cortical cells expressing Tbr1 and Ctip2 was disrupted, with a scattered distribution. The effect was similar, but minor, in later born Satb2-expressing cortical cells. NAC protected against LPS-induced neuron toxicity *in vitro* and counteracted pregnancy loss and alterations in thickness and lamination of the neocortex *in vivo*. Fetal loss and abnormal fetal brain development were due to LPS-induced ROS production. NAC is an effective protective agent against LPS-induced damage. This finding highlights the key therapeutic impact of NAC in LPS-caused abnormal neuronal laminar distribution during brain development.

Bacterial intrauterine infection affects pregnant women from zygote implantation to delivery and in the peripartum period, impacting both the fetal and newborn stages. Most commonly, microorganisms enter the uterus through the vagina. The infection is transmitted via the placenta to the fetus, which eventually causes preterm labor, prenatal death, and neurodevelopmental impairments^1^. Bacterial intrauterine infections play a major role in the etiology of preterm labor, with a 25–40% association^1^,^2^. Clinical studies show that intrauterine infection is associated with increased levels of proinflammatory cytokines in the amniotic fluid^3^ and umbilical cord plasma^4^. It is also associated with cerebral palsy^5^, schizophrenia^6^, and autism spectrum disorders^7^. Experimental models of intrauterine infection have revealed the role of the inflammatory response in the alteration of fetal neuronal morphology^8^, changes in oligodendrocyte precursors^9^, reduction of oligodendrocyte number, hypomyelination of brain^10^, and a decrease of dopaminergic and serotoninergic neurons in offspring^11^. All of these contribute to deficits in brain formation. Indeed, maternal infection induces changes in fetal brain developmental events, including neurogenesis, myelination, synaptogenesis, and neurite patterning^12^. The current therapy against infection is to use antibiotics^13^. However, antibiotics have limited success in preventing preterm labor or fetal inflammation and may cause undesirable side effects, such as an increase in neonatal deaths^14^,^15^. Cytokine suppressive anti-inflammatory drugs (CSAIDs) or non-steroidal anti-inflammatory drugs (NSAIDs) are relatively promising for use in preventing maternal infection, but some of these drugs may still increase susceptibility to infections by specifically inhibiting NF-κB-dependent immune responses^13^. Accordingly, a safer and more effective treatment needs to be developed.

Neuronal migration occurs primarily between the 12th and 24th week of gestation in humans and the 11th and 16th day in rodents^16^,^17^. Neurons pass the subplate and migrate into the cortical plate along the radial glial process starting from the subventricular zone with an inside out pattern that forms neocortical laminae^18^. This is a highly programmed process regulated by genetic factors, such as integration of reelin, Lis-1, and doublecortin^17^,^19^,^20^. Deficiency in these regulators disrupts neuronal migration and cortical laminar organization^21^, causing morphological abnormalities, such as schizencephaly, porencephaly, lissencephaly, macrogyria, and microgyria^22^. These lead to psychiatric and neurological disorders^19^,^23^,^24^. Intracervical LPS administration has been used to model clinical bacterial infection in pregnant animals in many studies. For example, maternal LPS application elevated fetal inflammatory cytokines in amniotic fluid, placenta, and brain^25^. It also changed fetal brain morphology^26^,^27^, altered the neurotransmitter system and behaviors^28^,^29^, and specifically decreased the levels of reelin and brain-derived neurotrophic factor (BDNF), all of which are related to brain development^30^,^31^. However, little is known about the effect of prenatal inflammation in early molecular and cellular regulation specific to neuronal laminar positioning. In the study described herein, we used an animal LPS model to evaluate the impact of a prenatal inflammatory response on the laminar organization of cortical neurons.

Intrauterine infection induces maternal systemic inflammatory responses, increases ROS generation, and decreases antioxidant enzyme concentration and activity in neonatal offspring^2^. ROS trigger chemokine production in inflammatory cells, enhancing the inflammatory process^32^. The increased oxidative stress due to ROS contributes to LPS-induced intrauterine fetal death, intrauterine growth restriction, and preterm labor^33^,^34^. N-acetyl-cysteine (NAC), a non-specific free radical scavenger and NF-κB inhibitor, reduces maternal serum LPS-induced oxidative stress and the proinflammatory cytokines TNF-α, IL-6, and IL-1β^35^,^36^,^37^. This provides protective effects against fetal death and preterm labor^33^, apoptotic white matter loss, and learning and behavioral deficits^38^,^39^. In this study, we examined whether NAC application protects fetal brain development from maternal LPS-induced oxidative damage. Indeed, we found that NAC reduced ROS generation in macrophages, which leads to an increase in neuronal survival *in vitro*. NAC reduced embryo death due to decreases in amniotic fluid and fetal intrauterine oxidative and inflammatory responses. Furthermore, NAC prevented LPS-caused aberrant brain morphology and laminar cytoarchitecture defects. Collectively, these data suggest that NAC is a potential therapeutic agent against bacterial intrauterine infection.

## Results

### NAC protected primary cortical neurons against LPS-induced neurotoxicity

**Figure 1:**
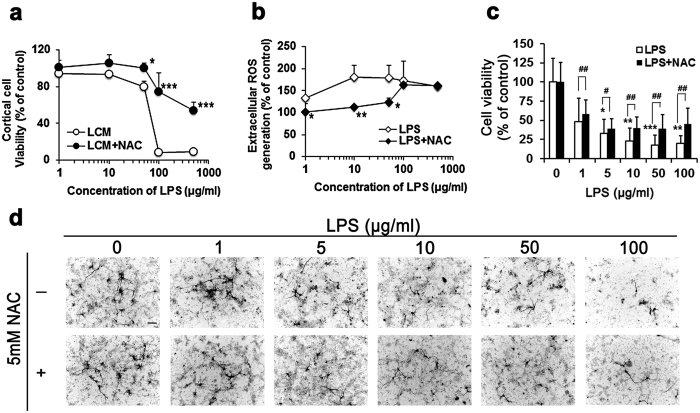
NAC rescued the LPS caused consequentially cortical neurotoxicity by inactivated macrophages secreting extracellular ROS. Primary cortical neurons were plated on 24 well plastic plates pre-coated with poly-D-lysine. At GD10, indicated LPS-conditioned media (LCM) with or without 5 mM NAC were applied for 48 hours followed by CytoTox- MTS Homogeneous Integrity Assay or fixation in 4% paraformaldehyde. Immunocytochemistry for MAP2 was conducted in fixed cells, and DAPI staining was performed to determine neuron survival. (**a)** Cm-H_2_DCFDA detection kit was used to determine the LPS-induced ROS generation in cultural medium of RAW264.7 cells. RAW264.7 cells were induced to release extracellular ROS in response to 24 h LPS exposure, and NAC decreased the level of ROS in lower dose of LPS treated group. *p < 0.05; **p < 0.01; ***p < *0.001* by ANOVA with Kruskal–Wallis test followed by Dunn’s multiple comparisons test. *n* = 3 assays, each performed in duplicate. (**b)** The cell viability decreased in a dose dependent manner and NAC inhibited the LPS-induced cell lost. *n* = 3 assays, each performed in duplicate. (**c)** Quantitative results from twenty different objective views were randomly selected from two to three independent experiments, and live cells were counted at each concentration. Significantly neuronal toxicity of LCM and dramatically rescued effect of NAC were observed. *Statistically different from vehicle condition without NAC. ^#^Statistically different from each LPS dosage compared to NAC. *p < 0.05; **p < 0.01; ***p < *0.001* by ANOVA with Kruskal–Wallis test followed by Dunn’s multiple comparisons test. *n* = 20. (**d)** Representative images of MAP2 positive neurons after LCM exposure in the presence/absence of NAC were generated from (**c**). Scale bar, 50 μm.

*In utero* events, such as intrauterine inflammation (also known as chorioamnionitis) trigger significant systematic inflammatory responses at the maternal-fetal interface and increase the incidence of inflammation in the central nervous system of the fetus^40^. Activated macrophages elevate cytotoxic molecules, such as free radical species and proinflammatory cytokines, which contribute to neurotoxicity in coculture systems^34^,^41^ and in a murine model^42^. NAC is a known ROS scavenger that not only ameliorates LPS-induced ROS generation and inflammatory effects in amniotic fluid and placenta, but also increases fetal viability and reduces white matter injury^36^,^43^. Therefore, we tested the protective effect of NAC against LPS-induced neurotoxicity in an *in vitro* model. Mouse macrophage cells, RAW264.7, were treated with various dose of LPS for 24 h, and the LPS-pretreated RAW264.7 culture medium (LCM) was collected and presented to heterogeneous primary cortical cells for 48 h. The RAW264.7 cells mimic simple maternal immune effectors, and the heterogeneous primary culture mimics the natural fetal brain with various neuronal and glial cells. LPS application increased LCM ROS levels up to 180% at 24 h (Fig. 1a). MTS assay results show heterogeneous cortical cell viability decreased in a dose-dependent manner (Fig. 1b). Cortical cultures were than immunostained with anti-MAP2 for localization analysis of only cortical neurons and to determine neuronal viability with DAPI staining. The result showed that neurons were killed 48 h after LCM treatment (Fig. 1c,d). Furthermore, we found that NAC blocked ROS production at lower LPS doses, but not at higher doses (Fig. 1a). Interestingly, cell viability was restored by NAC when applied with the LCM in heterogeneous cortical cultures at all doses (Fig. 1b). Neuronal number was restored in the presence of NAC, which suggests that the survival of neurons was enhanced (Fig. 1c,d).

### NAC inhibited maternal physiological changes in response to LPS exposure

**Figure 2:**
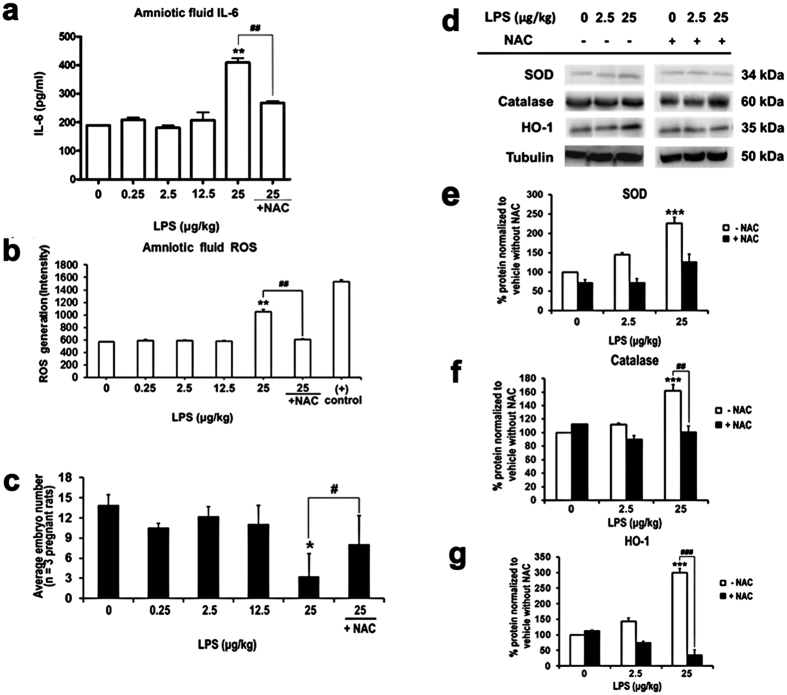
NAC prevented LPS caused ROS response and intra-uterine fetal death. **(a)** The IL-6 levels in amniotic fluid was detected by ELISA. The results show that intra-uterine LPS application caused significantly increased of amniotic IL-6 at highest does (25 μg/kg), and addition of NAC reduces this upregulation. *n* = 3 assays, each performed in duplicate. **(b)** Cm-H_2_DCFDA detection kit was used to determine the LPS-induced ROS generation in amniotic fluid. The highest concentration of LPS (25 μg/kg) dramatically induced ROS generation in amniotic fluid, and addition of NAC decreased this outcome. *n* = 3 assays, each performed in duplicate. **(c)** The number of embryo was decreased in response to LPS exposure. *n* values (number of pregnant female rats) for 0 μg/kg LPS = 11; 0.25 μg/kg LPS = 3; 2.5 μg/kg LPS = 5; 12.5 μg/kg LPS = 5; 25 μg/kg LPS = 5; 25 μg/kg LPS plus NAC = 5. **(d)** Representative Western blot of antioxidative markers in extract from five independent experiments is shown. **(e)** Quantitative analysis of SOD/tubulin band intensity shows an increase expression of SOD in a dose dependent manner, and the application of NAC eases the effect. *n* = 5. **(f)** Quantitative analysis of catalase /tubulin band intensity shows that high dose of LPS significantly induces catalase expression, and NAC hinders the act of LPS. *n* = 5. **(g)** Quantitative analysis of HO-1/tubulin band intensity shows an up-regulation of HO-1 in response to LPS, and addition of NAC dose-dependent reduces the HO-1 expression. *Statistically different from vehicle condition without NAC. ^#^Statistically different from each LPS dosage compared to NAC. *p < 0.05, **p < 0.01, ***p < 0.001 by ANOVA with Tukey-Kramer Multiple Comparisons Test. *n* = 5.

Our *in vitro* data show that NAC ameliorated LPS-induced cytotoxicity and ROS up-regulation. Therefore, we injected NAC and LPS maternally to analyze the effect of NAC in LPS-treated pregnant rats at GD14. Maternal organs, including spleen, placenta, and blood, were harvested to evaluate LPS-induced inflammation. We found that although all the parameters changed in the LPS group, there were no significant differences compared to controls at low doses of LPS (0.25–12.5 μg/kg) (Table 1). However, the weights of the spleen and placenta increased dramatically at a dose of 25 μg/kg LPS. At this dose, LPS not only reduced RBC number and hemoglobin (Hb) value, but also significantly increased WBC number (Table 1). We found that NAC rescued spleen and placenta weights, Hb level, and RBC and WBC numbers in LPS-treated samples. Another antioxidant, ascorbic acid, was less effective in most cases (Table 1). Furthermore, the concentrations of both amniotic fluid IL-6 (Fig. 2a) and ROS (Fig. 2b) were increased in the group that was treated with 25 μg/kg LPS. NAC reduced these concentrations to control levels. These data clearly show that NAC resolves LPS-stimulated maternal intrauterine oxidative and inflammatory responses.

### NAC counteracted the LPS-induced decrease in embryo number and up-regulation of antioxidative enzymes in embryonic brain

Since NAC mitigated the LPS-caused maternal intrauterine inflammation and amniotic fluid ROS concentration in our *in vivo* model, we then asked whether NAC could alleviate LPS-induced fetal death. We found that the number of embryos was significantly decreased in the 25-μg/kg LPS group. NAC restored embryo number to normal levels (Fig. 2c). In addition, ascorbic acid showed less protection (3.75 ± 3.5 embryos, *p* > 0.05 compared to the 25-μg/kg LPS group). Similarly, embryo weight was increased in the 25-μg/kg LPS group, and NAC returned this level close to control (Table 1). Previous studies suggest that LPS-induced oxidative stress and ROS contribute to fetal death^33^,^34^. NAC may have the capacity to attenuate LPS-induced cerebral white matter injury^37^. However, little is known about the role of NAC with regard to LPS-caused oxidative damage to the embryonic brain. Accordingly, we determined the levels of antioxidative enzymes in fetal brains. As shown in Fig. 2d to 2g, antioxidative enzymes (SOD, HO-1, and catalase) were significantly increased in fetal brains of the 25-μg/kg LPS group. In NAC-treated fetuses, these enzyme levels are normal. Our findings show that the antioxidative machinery is dramatically activated against ROS production in fetal brain even at 3 days after LPS application. These oxidative events were attenuated by NAC. This suggests that continuous oxidative stress can be relieved by NAC and thereby prevent fetal loss.

### NAC mitigated LPS-induced abnormal fetal brain cytoarchitecture

**Figure 3:**
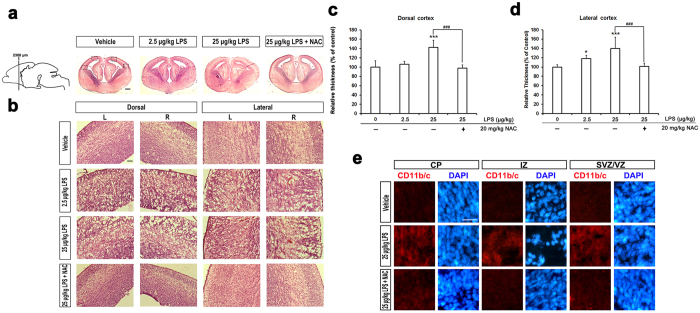
NAC lowered an increase in cortex thickness of fetal brain after prenatal exposure of LPS. **(a)** Coronal sections of indicated GD 18 rat embryonic brains were stained with H&E. It shows a significant swelling of brain especially in high dose of LPS-exposed fetus compared with vehicle exposed and NAC treated fetus. Scale bar, 500 μm. **(b)** The dorsal and lateral views of left (L) and right (R) cortex are shown as the position of open frame in (**a**). The turgid nucleus, vacuous configuration, and vascular proliferation were appeared in LPS-exposed fetus compared with PBS-exposed and NAC treated fetus. Scale bar, 50 μm. **(c)** Quantifications of the differences in the thickness of the dorsal cortex. **(d)** Quantifications of the differences in the thickness of the lateral cortex. (**c**) and (**d**) Five embryo brains were randomly selected from at least three different pregnant rats. In each brain, two coronal section near the site around 2380 μm from the front of olfactory bulb as indicated in (**a**) were analyzed. Both dorsal and lateral cortical area of LPS-exposed fetus was significantly larger than in PBS-exposed and NAC treated animals. Histograms represent the means ± SD. *Statistically different from vehicle condition without NAC. ^#^Statistically different from each LPS dosage compared to NAC. *p < 0.05; ***p < 0.001 by ANOVA with Kruskal–Wallis test followed by Dunn’s multiple comparisons test. **(e)** Coronal sections of indicated GD18 rat embryonic brains were immunostained for CD11b/c (microglia marker) and DAPI (nuclei). Representative images from three independent experiments are shown in cortical plate (CP), intermediate zone (IZ), and sub-ventricular and ventricular zone (SVZ/VZ). Microglia were activated in LPS treated animals, which were reduced in the presence of NAC. Scale bar, 50 μm.

Previous studies offer inconsistent results regarding changes in fetal brain morphology after prenatal immune activation. For example, Carpentier and colleagues found that cortical thickness was decreased in LPS-treated animals relative to saline controls^30^. However, increased thickness of the cortical plate and hippocampus was reported by Ghiani and colleagues^44^. This discrepancy may be due to differences in infection models, target animal species, and infection protocol and time^12^. To evaluate the effect of LPS on the morphology of fetal brain in our *in vivo* model, we analyzed coronal sections of LPS-exposed fetal brains using H&E staining. LPS caused brain swelling particularly in the high-LPS-dose group (Fig. 3a). Application of NAC ameliorated the effects. We examined frontal neocortex thickness in both dorsal and lateral regions (location shown in Fig. 3a marked with open rectangle). The data show a significant 20% increase in the depth of lateral and dorsal neocortex in the group treated with a high dose of LPS (Fig. 3c,d). Application of NAC reduced these effects, and the thickness of the neocortex in this group was comparable to control levels (Fig. 3c,d). Furthermore, we found that the cellular structure displayed a swollen nucleus and unregulated vascular proliferation in response to LPS (Fig. 3b). The data imply a white matter injury morphology in the LPS groups^39^. These characteristics are obvious at 25 μg/kg LPS, and NAC inhibited these changes. In addition, a previous report shows that glial activation is associated with maternal *E. coli*-induced white matter injury^10^. We then analyzed the activation of microglia by immunostaining for CD11b/c, a microglia marker. As shown in Fig. 2e, 25 μg/kg LPS induced microglial activation in the fetal cortical plate (CP), intermediate zone (IZ), and subventricular and ventricular zones (SVZ/VZ). These effects were diminished by NAC exposure.

### NAC counteracted the LPS-induced aberrant lamination of embryonic cortex

**Figure 4:**
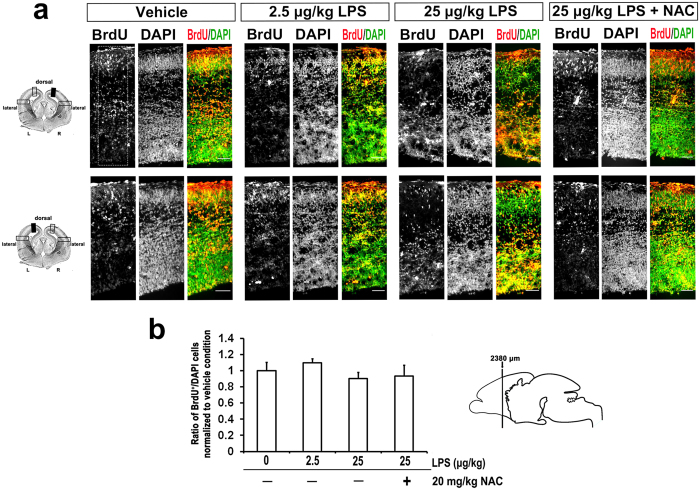
Maternal LPS and NAC exposure had no effects on embryonic neurogenesis. BrdU (50 mg/kg/day) were injected intradermally from GD 15 to GD 18 after exposure to LPS. Animals were sacrificed at GD 18 and immunohistochemistry was followed. Coronal sections of indicated GD 18 rat embryonic brains were immunostained for BrdU and DAPI. Merged image is composed of BrdU (red) and DAPI (green). **(a)** Representative images of the sections at cortical dorsal area from three independent experiments are shown. BrdU^+^ cells were displayed closely to ventricles in high doses of LPS treated group compared to vehicle exposed group, which BrdU^+^ neurons were localized mostly in cortical plate. The effects of NAC returned the abnormal laminar characteristic to control levels. Scale bar, 50 μm. **(b)** displays the quantitative results of BrdU^+^ and DAPI^+^ cells in the indicated dorsal area of cortex (dotted square in (**a**)). Four embryo brains were randomly selected from at least three different pregnant rats. In each brain, two coronal sections near the site around 2380 μm from the front of olfactory bulb were analyzed. Ratio of BrdU^+^/DAPI in each condition was normalized to the PBS-exposed group. LPS treatment had no effects on the neurogenesis, determined by ANOVA followed by Tukey-Kramer Multiple Comparisons Test.

**Figure 5:**
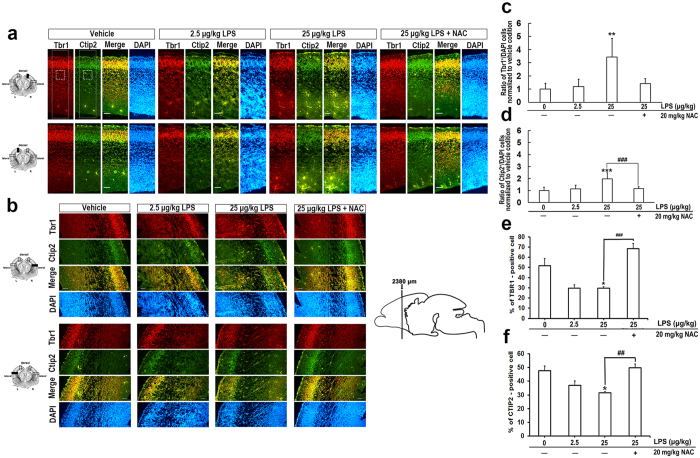
NAC mollified the abnormal cortical lamination in the developing embryonic brain after maternal exposure to LPS. Coronal sections of indicated GD 18 rat embryonic brains were immunostained for Tbr1, Ctip2 and DAPI. Tbr1 indicates the cells in cortical layer VI, while Ctip2 labels layer V cells. Merged image is composed of Tbr1 (red), Ctip2 (green). DAPI (blue) represents each nuclei. Five embryo brains were randomly selected from at least three different pregnant rats. In each brain, two coronal sections near the site around 2380 μm from the front of olfactory bulb were analyzed. Representative images of the sections at cortical **(a)** dorsal and **(b)** lateral areas are shown. Both Ctip2^+^ and Tbr1^+^ cells are scatteredly distributed in LPS treated group comparing to vehicle exposed and NAC treated fetus. Scale bar, 50 μm. **(c)** and **(d)** display the quantitative results of the small dashed square. The dorsal area of cortex was divided into 3 equivalent bins, with the border between bin 1 and bin 2 corresponding to the edge of intermediate zone or the area just fall off the cortical plate, as the dotted area. Ratio of Tbr1^+^/DAPI and Ctip2^+^/DAPI in each condition was normalized to the vehicle. Application of LPS results in majority of theTbr1^+^ and Ctip2^+^ cells remaining inside the intermediate zone, and meantime NAC returned the ratio to vehicle condition. *Statistically different from vehicle condition without NAC. ^#^Statistically different from each LPS dosage compared to NAC. **p < 0.01, ***p < 0.001 by ANOVA followed by Bonferroni multiple comparisons test or Dunn’s multiple comparisons test. **(e,f)** display the quantitative results of the large dotted square, as same as the area counted for BrdU. Number of Tbr1^+^ or Ctip2^+^ cell was normalized to the number of DAPI^+^ cell. LPS caused significantly less Tbr1^+^ and Ctip2^+^ cells expressed, and meantime NAC had rescue effect. *Statistically different from vehicle condition without NAC. ^#^Statistically different from each LPS dosage compared to NAC. *p < 0.05, **p < 0.01, ***p < 0.001 by ANOVA followed by Tukey-Kramer Multiple Comparisons Test.

**Figure 6:**
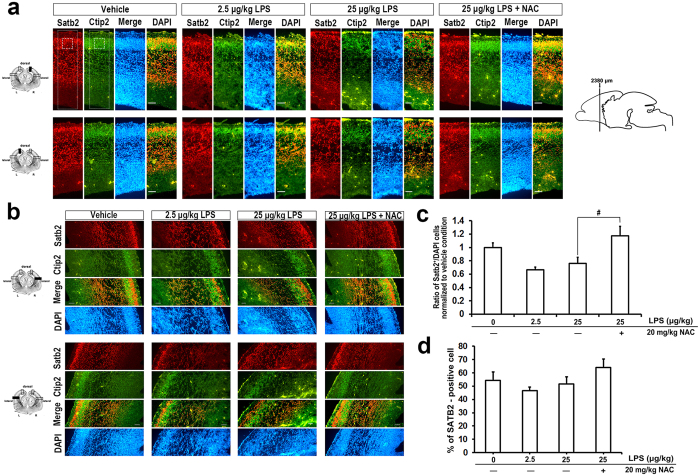
LPS induced impairment in neural development was recovered by application of NAC. Coronal sections of indicated GD 18 rat embryonic brains were immunostained for Satb2, Ctip2 and DAPI. Satb2 expressing neurons are mainly localized in layer II/III and moderately in layer VI, while Ctip2 labels layer V cells. Merged image is composed of Satb2 (red), Ctip2 (green). DAPI (blue) represents each nuclei. Five embryo brains were randomly selected from at least three different pregnant rats. In each brain, two coronal sections near the site around 2380 μm from the front of olfactory bulb were analyzed. Representative images of the sections at cortical **(a)** dorsal and **(b)** lateral areas are shown. Comparing to vehicle-exposed group, Ctip2^+^ cells expressed extendedly in LPS treated group, significantly in higher dose. Satb2^+^ neurons were more dispread and disorganized after exposure to high dose of LPS. The effects of NAC returned abnormal cortical lamination characteristics to control levels. Scale bar, 50 μm. **(c)** displays the quantitative results that the quantitative results of the small dashed square. The dorsal area of cortex is divided into 9 equivalent bins, with the border between bin 1 and bin 2 corresponding the cortical plate as the dotted area. Ratio of Satb2^+^/DAPI in each condition is normalized to the vehicle. LPS caused lesser Satb2^+^ cells expressed in cortical plate, and application of NAC effectively restored the location of laminar Satb2 distribution. ^#^*p* < 0.05 by ANOVA followed by Tukey-Kramer Multiple Comparisons Test. **(d)** displays the quantitative results of the large dotted square, as same as the area counted for BrdU. Number of Satb2^+^ cell was normalized to the number of DAPI^+^ cell. There is no significant differences in the total number of Satb2^+^ cell among groups. ^ns^*p* > *0.05* by ANOVA followed by Tukey-Kramer Multiple Comparisons Test.

Prenatal exposure to LPS causes a decrease in neurogenesis, myelination deficits, and abnormal neuronal morphology in developing brains^12^. As shown in our *in vivo* model, NAC could prevent LPS-induced damage to fetal brain morphology (Fig. 3). We next asked whether NAC can improve LPS-induced reductions in neurogenesis. When the ratio of bromodeoxyuridine (BrdU)-positive cells in each condition was assessed after normalizing to the vehicle, we found no difference in neurogenesis with or without LPS treatment (Fig. 4). Moreover, application of NAC did not affect the number of BrdU-labeled cells (Fig. 4). However, we found BrdU-positive cells distributed close to the ventricles at high doses of LPS compared to the vehicle group, for which BrdU-positive cells are localized mostly in the cortical plate. NAC treatment prevented these abnormal characteristics. Mammalian brains have a 6-layer neocortex; each layer has a specific function and forms appropriate local and long-distance connections. Laminar formation of the neocortex occurs via an inside-out pattern, with the oldest neurons in the deepest layer (layer VI), and this process is highly programmed by genetic control during the embryonic period^20^,^45^. Specific genes such as Tbr1 (T-box brain 1), Ctip2 (COUP-TF interacting protein 2), and Satb2 (special AT-rich sequence binding protein 2) play important roles at each step during differentiation, and disruption leads to profound cortical malformation^20^,^45^. Carpentier and colleagues have shown that there are stereotypical alterations in cortical patterning in LPS-exposed mice^30^, although this has not yet been replicated in other studies. Even less is known about how NAC influences LPS-affected neural laminar positioning. To examine this, we used markers [Tbr1 (layer VI), Ctip2 (layer V), and Satb2 (layers II/III and VI)] to label different cortical layers in coronal fetal brain sections. The number and distribution of cells displaying each marker were quantitated. We found that LPS altered the expression pattern of cortical lamination markers. Satb2, Tbr1, and Ctip2 expression were disrupted and distributed unevenly in the LPS-treated group (Figs 5a,b and 6a,b). NAC treatment reduced the layer disorganization. Furthermore, the quantitative results show a disrupted laminar pattern of Tbr1- and Ctip2-labeled cells, which was diminished by NAC (Fig. 5a,c). There was a trend in the perturbation of the laminar position of Satb2-labeled cells, and NAC reversed the trend to that of controls (Fig. 6c). We calculated the total number of Satb2-, Tbr1-, and Ctip2-labeled cells in a unit width of cortex. Results show that cells marked with Tbr1 and Ctip2 were significantly decreased in the 25-μg/kg LPS-exposed group (Fig. 5e,f). Satb2 was not affected (Fig. 6d). The reductions of Tbr1- and Ctip2-labeled cells were restored by NAC. These data suggest that LPS-induced ROS damage dramatically affects lamination in the fetal cortex.

### Prolonged effects of LPS-induced intrauterine inflammation in fetal brain

**Figure 7:**
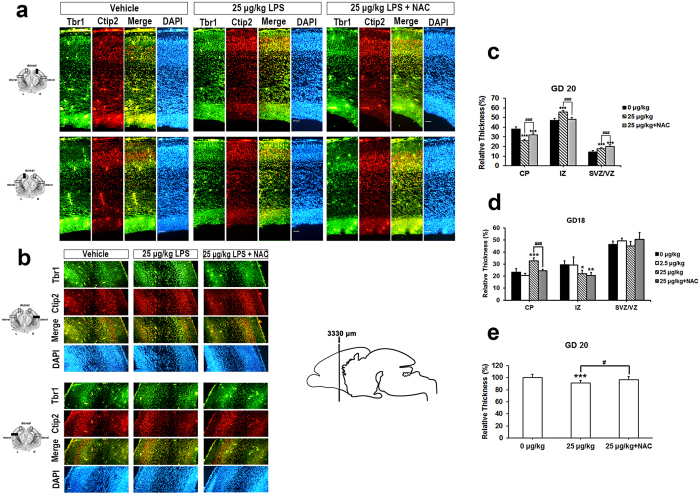
NAC restored the sustainable effects of LPS on the late embryonic brain development. Coronal sections of indicated GD 20 rat embryonic brains were immunostained for Tbr1, Ctip2 and DAPI. Five embryo brains were randomly selected from at least three different pregnant rats. In each brain, two coronal section near the site around 2380 μm from the front of olfactory bulb were analyzed. Representative images of the sections at cortical dorsal **(a)** and lateral (**b)** areas from three independent experiments are shown. Tbr1^+^ cells are distinguished from Ctip2^+^ cells in vehicle condition, but interlaced at LPS treated group. Scale bar, 50 μm. **(c)** The portion of dorsal cortical plate (CP), intermediate zone (IZ), and sub-ventricular and ventricular zone (SVZ/VZ) relative to total thickness from the edge of ventricle to the top surface of the GD 20 brain were determined. LPS causes a significant decrease in CP thickness compared to vehicle condition. ***p < 0.001 by Unpaired t test in each zone. **(d)** The portion of dorsal cortical plate (CP), intermediate zone (IZ), and sub-ventricular and ventricular zone (SVZ/VZ) relative to total thickness from the edge of ventricle to the top surface of the GD 18 brain were determined. CP portion was dramatically increased in LPS treated group, which was returned to vehicle condition in the presence of NAC. *** or ^###^*p* < 0.001 by ANOVA followed by Tukey-Kramer Multiple Comparisons Test or Dunn’s Multiple Comparisons Test. **(e)** Quantifications of the differences in the thickness of the dorsal cortex. Histograms represent the means ± SD. *n* = 10. ***p *<* *0.001* by Student’s-t Test.

To determine whether the LPS-induced abnormal laminar pattern in developing brains is a temporally delayed action or an irreversible outcome, we examined LPS-treated fetal brains at 20 d of gestation with the Tbr1 and Ctip2 cortical laminae layer markers. We found that Tbr1- and Ctip2-labeled cells overlapped each other in the LPS-treated group (Fig. 7a). In the control group, layer V is clearly distinguished from layer VI (Fig. 7a). This distinct layering is normal at later stages of brain development^17^,^20^. We also calculated the proportion of cortical plate, intermediate zone, and subventricular/ventricular zone to the thickness of the whole cortex at GD20. The result shows that, compared to controls, the cortical plate is thinner in LPS-exposed fetal brains, whereas the intermediate zone and subventricular/ventricular zone are thicker (Fig. 7b). However, the trend is different in GD18 fetal brains with the proportion of cortical plate increased and intermediate zone reduced in the LPS-treated group (Fig. 7c). No significant difference was found in the subventricular/ventricular zone. NAC resolved the abnormal distribution to control levels, except in the intermediate zone (Fig. 7c). Interestingly, the total thickness of the fetal cortex was decreased by LPS treatment at GD20 (Fig. 7d), but increased at GD18 (Fig. 3c,d). These data suggest that maternal infection causes an irreversible shift in the architecture of the fetal brain during development.

## Discussion

Intrauterine infection induces serious complications in both the mother and the fetus. For the fetus, one major complication is preterm labor with a high risk for fetal death. Organ infections or abnormal development are common in premature infants^1^,^44^. Perinatal brain injury is also a significant clinical problem caused by intrauterine infection due to the inflammatory mediators, which further lead to acute or chronic brain disease^46^. These inflammatory mediators include ROS and cytokines. Previous studies have shown increased ROS production and associated antioxidant events in preterm infants^2^. Systemic administration of LPS mimics maternal infection and induces macrophage activation^41^,^42^; ROS and proinflammatory cytokine secretion contribute to neurotoxicity^41^,^42^,^47^. Boksa and colleagues have compiled reports from 2001 to 2009 on the effects of LPS on the fetal brain, but controversy remains^12^. Antibiotics, NSAIDs and CSAIDs, are used to treat maternal infections, but have some undesirable side effects^13^. The development of innovative therapies therefore remains essential for progress in the prevention and treatment of intrauterine infections. In our study reported herein, using both *in vitro* and *in vivo* models, we demonstrated that NAC reduced LPS-induced oxidative damage in cultured neurons and in fetal brain, counteracted an irreversible abnormality in neuron laminar positioning during development, and decreased fetal death.

Macrophages are one of the primary maternal immune cells that respond to intrauterine infection. LPS triggers ROS elevation in macrophages, which contributes to the inflammation and fetal complications^41^,^42^. Increased ROS also result in LPS-induced intrauterine fetal death, intrauterine growth restriction, and preterm labor^33^,^34^,^48^,^49^. In the present study, we did not observe LPS-induced fetal growth restriction after prenatal exposure to LPS. This may be due to the lower LPS dose (25 μg/kg) at a later embryonic day, 14 d gestation, compared with other findings^12^. However, we did observe significantly decreased embryo number (Fig. 2c) and increased ROS and IL-6 levels in amniotic fluid, consistent with previous findings^37^,^48^,^50^. Furthermore, treatment with NAC significantly reduced embryo loss and amniotic fluid ROS and IL-6 levels (Fig. 2a–c), which is supported by other reports showing that NAC improved survival of offspring after LPS injection in rat dams at GD16-18^36^,^51^, provided protection against fetal death and preterm labor induced by maternal inflammation^33^, and lowered the inflammatory cytokine response in amniotic fluid and placenta in rats^35^,^37^. A previous publication reported that NAC has potential life-threatening effects on a fetus that has been exposed to endotoxins, which worsens LPS-induced fetal hypoxemia and hypotension^52^. However, our data show no reduction of hemoglobin and erythrocyte levels when NAC is administered (Table 1). Furthermore, da Rosa and colleagues have reported that NAC diminishes liver inflammation and apoptosis in an intermittent hypoxia model^53^. This discrepancy may be due to differences in dosage and injection location of NAC. Probyn and colleagues directly gave 50 to 200 mg/5h to the fetus, while in other studies NAC was administered intraperitoneally to pregnant animals at doses of 50 to 300 mg/kg^49^,^51^,^54^. In our study, one intraperitoneal injection of a low dose of NAC (20 mg/kg) was used, which may reduce the incidence of LPS-induced hypoxemia or hypotension. Moreover, a clinical study of NAC has been done for bacterial intrauterine infection^55^. The results of this study confirm that NAC may be a potential therapeutic approach for the prevention and treatment of maternal infection.

Our study shows that LPS caused brains to swell and the cortex to thicken with a turgid nucleus and vacuous appearance of fetal brains in rats after prenatal exposure to 25 μg/kg LPS. The etiology of the vacuous appearance of fetal brains in the LPS-treated group may be LPS-induced injury to oligodendrocyte precursors in the developing brain. This may further lead to white matter injury, with consequences for cerebral palsy and related demyelinating diseases in premature infants^9^,^10^,^37^. Increased cortex volume or thickness is a consistent result supported by others^27^,^44^, but reduced cortical volume^26^ or thickness^30^ is not consistently observed. These discrepancies may be due to differences in LPS dosage and the time and brain region of post-injection observation. Indeed, we found that cortical thickness was reduced at 20 d of gestation (Fig. 7d). A portion of the cortical plate displayed a consistent trend with regard to the thickness of the cortex at both GD18 and GD20 (Fig. 7b,c), implying a preferential impact of LPS in that area. The ratio of intermediate zone is more likely correlated with changes in the cortical plate in a contrasting relationship. We found no significant difference in the percentage of subventricular/ventricular zone at GD18, but did notice an effect at GD20, which may be related to LPS-induced gliosis or migration failure as discussed below. The alteration of cortex or cortical plate thickness may be due to the initial proliferation and activation of microglia, which subsequently decreases or returns to normal^12^,^56^. Indeed, we found that microglia are activated in the presence of LPS (Fig. 3e) at GD18, but not at GD20 (data not shown). However, a recent finding shows no increase in microglial cell activation in fetal brain in a polyinosinic:polycytidylic acid infection model^57^. Another possible explanation is that this may be due to an increase in apoptosis after maternal LPS exposure^12^. All of the aberrant LPS-induced changes in brain morphology were attenuated by administration of NAC (Figs 3 and 7), which indicates the involvement of oxidative stress^37^,^51^.

The development of a 6-layer neocortex starts at GD11.5 in rodents and 12 weeks of gestation in humans^17^,^24^. The process is coordinated by specialized genes, such as reelin, which program neurogenesis and neuronal subtype specification^17^,^19^,^20^. Satb2, Ctip2, and Tbr1 are markers of lineage-committed progenitors in specific layers as well as transcription factors necessary for the development of the corresponding laminae^20^,^45^. In Fig. 4a–d, we show a significant disruption in laminar positioning of Ctip2- and Tbr1-labeled cells at GD18 and a scattered distribution relative to each other. However, Satb2-labeled cells displayed only a trend of arrested lamination and a slight shift in position relative to Ctip2. This may be due to the time of LPS injection, when Ctip2 and Tbr1 are notably expressed, but Satb2 is not. Tbr1 and Ctip2 are expressed in deep laminae during neurogenesis and cell migration, but away from Satb2-regulated events (known as an inside-out development of patterning)^17^. Indeed, neurogenesis of Ctip2^+^ and Tbr1^+^ cells was significantly decreased (Fig. 5e,f), but Satb2^+^ cell proliferation showed no notable differences (Fig. 6d) either with regard to neurogenesis or in the whole cortex (Fig. 3b). LPS-induced disruptions in cell distribution and neurogenesis in laminae layers may have also contributed to the reduction in thickness of the cortical plate at GD20 (Fig. 7b). These findings strongly suggest that LPS induced laminar imbalance along the cortical layer patterning axis and also caused a decrease in neurogenesis^30^. However, there was no difference in the number of BrdU^+^ cells (Fig. 4), which indicated proliferating neurons or glial cells^58^,^59^. This may be due to the decreased neurogenesis counteracting the LPS-induced BrdU-labeled microglia. Satb2 generates callosal projections in layers II to IV and makes subcortical connections^60^,^61^. Tbr11 is highly expressed in preplate and layer VI neurons and makes corticocortical projection neurons^62^. Ctip2 is located in layers V and VI and promotes corticospinal motor neuron projection^61^,^63^. The significant reduction in specific layers of the cortical plate leads to an abnormality that alters axon projections. Such alterations in neocortical development may demonstrate how adverse perinatal events predispose the damage in the developing brain toward later behavioral abnormalities^64^,^65^,^66^. Importantly, all lamination deficits were prevented by NAC. Other factors should not be ruled out in the case of morphological changes in the developing fetal brains. For example, a reduction in brain-derived neurotrophic factor or nerve growth factor caused by LPS affects neurogenesis in the fetal brain^31^; reelin expression is decreased in fetal brain^44^; lower activation and expression of Pax6 in cortical progenitors was found in the polyriboinosinic-polyribocytidylic acid infection model^67^.

We found the level of GSH, SOD, and catalase in fetal brain increased in a LPS dose-dependent manner (Fig. 2d–f). These findings indicate that endogenous tissue antioxidants respond to the increased oxidative levels induced by LPS, also noted in a previous study^68^. NAC is an endogenous thiol-containing amino acid that scavenges ROS by interactions with its thiol redoxing group. We show that NAC promoted detoxification and prevented redox failure. NAC is able to cross the placenta and the blood-brain barrier, where NAC preserves peroxisomes, rescues cerebral oligodendroglial precursor cells, restores myelination^37^, decreases caspase 1 and 3 expression in fetal brains with hypoxia ischemia^69^, increases the glutathione level in fetal liver^33^, and regulates antioxidant machinery to restore it to normal levels^70^. However, our results showed that LPSincreased the expression level of antioxidant enzymes three days after LPS injection (Fig. 2d–g), similar to other published reports^71^,^72^. This suggests that LPS evoked temporally specific oxidative stress, which could be regulated by NAC.

Herein we show that NAC attenuated deleterious effects caused by LPS, including fetal loss, oxidative and inflammatory responses, and abnormal neocortical lamination. This strongly suggests that NAC is a potential neuroprotective factor against maternal bacterial infection.

## Materials and Methods

### Animals and chemicals application

All experiments were conducted in accordance with the principles of animal care and experimentation in the Guide for the Care and Use of Laboratory Animals. The Institutional Animal Care and Use Committee of the Chung Yuan Christian University approved the use of animals in this study. Six to eight weeks old Sprague Dawley rats of both sexes were purchased from BioLASCO Taiwan. The rats were put as a pair for mating once a pregnant rat was planned for the experiment. For LPS treatment, pregnant rats were injected intraperitoneally with 0, 0.25, 2.5, 12.5 or 25 μg/kg/day LPS (Sigma-Aldrich) with or without pretreated 20 mg/kg/day NAC or ascorbic acid 1 hour before LPS at GD14. For bromodeoxyuridine (BrdU) labeling, pregnant rats were injected with a single dose of 5-bromodeoxyuridine solution (50 mg/kg/day in PBS) (Sigma-Aldrich) three days from GD 15 to GD 17. At GD 18 or GD 20, the mother was sacrificed with CO_2_ inhalation, the blood, liver, spleen and embryo was collected immediately for analysis.

### Cell culture and treatment

The mouse macrophage-like RAW264.7 cell line was obtained from the American Type Culture Collection (ATCC, Rockville, MD). They were grown in Dulbecco’s modified Eagle’s medium (DMEM) supplemented with penicillin, streptomycin and 10% FBS in a humidified atmosphere with 5% CO_2_ at 37 °C. Cells were subcultured when confluence reached to 90%. For primary cortical cell culture, the cultures were prepared from cortices of rat embryos of both sexes at GD 18. Cortex was dissociated and the cells were plated on glass coverslips (12 mm in diameter) coated with poly-D-lysine at a density of 1200–1600 cells/mm^2^. The cells were cultured in Neurobasal media supplemented with B27, penicillin, streptomycin, and GlutaMax (Invitrogen, Carlsbad, CA) for 10 days prior to the treatments. For LPS application, RAW264.7 cells were treated with LPS for 24 h. The LPS-pretreated RAW264.7 cultured media (LCM) thereafter was collected and placed into the cortical cultures for additional 48 h.

### Analysis of extracellular and amniotic ROS production

ROS detection studies were performed using a Cm-H_2_DCFDA detection kit (Invitrogen). After exposure to LPS ± NAC, the cultured media LCM or the amniotic fluid was collected and pipetted into 96-well plate and mixed with 10 μL Hank’s Buffered Salt Solution (HBSS) containing Cm-H_2_DCFDA reagent (25 μM final concentration in each well). ROS concentration was determined immediately at fluorescence 485/530 nm by using a microreader. The ROS production values were normalized and presented as percentage of the control.

### Amniotic fluid IL-6 detection

IL-6 was evaluated using a Rat IL-6 ELISA Kits (R&D Systems, Minneapolis, MN, USA), following the manufacturer’s instructions. The levels of fluid IL-6 was determined by comparison to a standard curve, which was prepared by analyzing 2-fold serial dilutions of each cytokine.

### Determination of cytotoxicity in cortical cells

Cytotoxicity was determined by the commercial CytoTox- MTS Homogeneous Integrity

Assay Kit (Promega, Madison, WI). The live cortical cells were determined with quantifying their mitochondrial enzyme activity via reductive conversion of the tetrazolium salt MTS to a soluble formazan dye. The amount of the dye was measured spectrophotometrically at absorbance 490 nm. Values were normalized and expressed as percentage of the control.

### Neurons viability

The LPS ± NAC treated cortical cells were fixed with 4% paraformaldehyde in PBS for 15 min at room temperature, followed by PBS washes. The fixed neurons were then immunostained with mouse anti-MAP2 (neuron marker) (1:500, Abcam, Cambridge, MA). MAP2 positive neurons were then visualized with FITC or Alexa Fluor 488 anti-mouse secondary antibodies (Jackson ImmunoResearch Inc.) under a 20X objective. Nuclei were stained using DAPI (Sigma) to determine cell survival. At least twenty different objective views were randomly selected from two to three independent experiments. The experimenter was blinded to the condition when taking images and counting. Neurons with positive MAP2 immunostaining, and even and intact DAPI staining were considered alive.

### Immunoblot

The tissue lysates were prepared in SDS containing sample buffer, and equal volumes of lysates were separated by 12% SDS-PAGE gel. Proteins were transferred to nitrocellulose membrane and the blots were probed with the following primary antibodies: mouse anti-SOD (1:1000, Abcam); rabbit anti-catalase (1:2500, Abcam); mouse anti-HO-1 (1:10000, Abcam), and mouse anti-α-tubulin (1:5000, Abcam). Appropriated HRP-conjugated secondary antibodies were then applied to the blots. Blots were visualized by enhanced chemiluminescence (Promega™ ECL Western Blotting Substrate) and analyzed on the Odyssey Infrared imaging system (LI-COR Biosciences) (Lincoln, NE).

### Immunohistochemistry and analysis of fetal brain development

The fetal brain were harvested at GD 18 or GD 20 in some experiments, and was perfused and fixed in 4% paraformaldehyde for at least 12 h, sunk in 30% sucrose/PBS and embedded in OCT and frozen at −80 °C. The fixed samples were sectioned at 10 μm with a cryostat. A GD 18 fetal brain was carried out approximately 780 slices and a GD 20 fetal brain was approximately 1090 slices. In each quantification, four to five fetal brains from different pregnant rats were collected. In each brain, two coronal sections near the site, around 2380 μm for GD18 and 3330 μm for GD20, from the front of olfactory bulb were analyzed. The sections were dried overnight. Nonspecific reactivity was blocked and sequentially incubated with primary rabbit anti-TBR1 (1:500, Abcam), rat anti-Ctip2 (1:500, Abcam), mouse anti-SATB2 (1:400, Abcam) and mouse anti-BrdU (1:500) (Sigma-Aldrich).The slides were transferred to secondary antibodies labeled with Alexa Fluor 488 and 594 (Jackson ImmunoResearch Inc.) and covered with an anti-fade mounting media with DAPI. Images were observed on an IX51 Olympus Microscope. For lamination analysis, dorsal area of coronal cortical section was analyzed using NIH Image J software based on a previous report^73^ with modifications. Briefly, the depth of cortex was divided into 3 equivalent bins, with the border between bin 1 and bin 2 corresponding to the edge of intermediate zone or the area just fall off the cortical plate. The ratio of Ctip2^+^ or Tbr1^+^ cells to DAPI staining in a square area (1.4 × 1.4 μm^2^) at the border between bin 1 and 2 was analyzed. The ratio of Ctip2^+^/DAPI and Tbr1^+^/DAPI in each condition is normalized to the vehicle. A similar method was performed for Satb2^+^/DAPI cells counting. The dorsal area of cortex is divided into 9 equivalent bins, with the border between bin 1 and bin 2 corresponding the cortical plate. Ratio of Satb2^+^/DAPI in each condition is normalized to the vehicle. The depth of cortex was measured along a line orthogonal to the superficial pia and callosal surfaces. At least five images from two to three independent experiments were counted at each group. For neurogenesis, the ratio of BrdU^+^/DAPI, Satb2^+^/DAPI, Ctip2^+^/DAPI or Tbr1^+^/DAPI cells were counted at an oblong area of dorsal cortex with fixed width (108.1 μm) over entire thickness of cortex. The ratio of cortical plate, intermediate zone, and subventricular/ventricular zone to the depth of cortex was analyzed. At least five images from two to three independent experiments were counted at each group.

### H&E (hematoxylin and eosin) staining and analysis

The coronal sections of LPS-exposed fetal brain were stained with H&E staining based on published report^74^ with some modification. Briefly, the slides were post-fixed with 4% PFA, followed by distilled water washes. Slides were stained with Hematoxylin for 5 min and washed out with distilled water and 95% ethanol. Eosin were then applied for 3 min. Sequential dehydration steps (50 to 100% ethanol and xylene) were followed and mounting solution was applied. The slides were visualized by general phase contrast microscope. The thickness of dorsal or lateral neocortex were analyzed by NIH Image J software following the method based on previous report^44^. Dorsal neocortex measurements were obtained from presumptive motor area above the middle body of the corpus callosum. Total dorsal cortical thickness were measured along a line orthogonal to the superficial pia and callosal surfaces. Lateral cortex measurements were obtained from the presumptive boundary in between central neocortex and insular cortex. Total lateral cortical thickness were measured along a line orthogonal to the superficial pia and ventricle surfaces between striatal subventricular zone and neocortical subventricular zone. Five embryo brains were randomly chosen from at least three different pregnant rats in each condition. In each brain, two coronal sections near the site around 2380 μm from the front of olfactory bulb were analyzed. There were approximately 780 sections in a GD18 fetal brain.

### Statistics

Statistical significance (*p* < 0.05) was determined using ANOVA followed by the appropriate post hoc test or Student’s t-Tests using GraphPad InStat software.

## Additional Information

**How to cite this article**: Chao, M.-W. *et al*. N-acetylcysteine attenuates lipopolysaccharide-induced impairment in lamination of Ctip2-and Tbr1- expressing cortical neurons in the developing rat fetal brain. *Sci. Rep.* **6**, 32373; doi: 10.1038/srep32373 (2016).

## Figures and Tables

**Table 1:** Maternal and embryo physiological conditions changed in response to LPS plus or minus NAC exposure on GD 18.

Parameters	Vehicle	LPS (0.25 μg/kg)	LPS (2.5 μg/kg)	LPS (12.5 μg/kg)	LPS (25 μg/kg)	LPS (2.5 μg/kg) + NAC	LPS (25 μg/kg) + NAC	LPS (25 μg/kg) + Asc
Spleen Weight (%)	100 ± 14.8	82 ± 0.1	106 ± 12.6	107 ± 2.5	202 ± 21.7***	108 ± 1.9	112 ± 9.8^#^	154 ± 29.1***
Placenta Weight (g)	0.35 ± 0.06	0.31 ± 0.04	0.36 ± 0.05	0.32 ± 0.05	0.42 ± 0.09*	0.38 ± 0.05	0.32 ± 0.06^###^	0.41 ± 0.10^###^
Placenta Area (cm^2^)	1.36 ± 0.10	1.31 ± 0.11	1.34 ± 0.11	1.29 ± 0.13	1.31 ± 0.11**	1.30 ± 0.07	1.31 ± 0.16	1.34 ± 0.20
Embryo Weight (g)	0.94 ± 0.13	0.95 ± 0.13	0.95 ± 0.16	0.91 ± 0.09	1.09 ± 0.22**	0.99 ± 0.13	0.94 ± 0.13	0.90 ± 0.08***^,#^
Hemoglobin (g/dL)	16.4 ± 2.68	15.3 ± 0.52	14.0 ± 0.93	12.5 ± 5.67	13.5 ± 2.96***	14.5 ± 0.84	15.1 ± 1.05^##^	11.8 ± 1.60
RBC (10^9^/mL)	6.48 ± 2.27	6.59 ± 0.57	4.78 ± 1.26	4.66 ± 0.60	3.62 ± 1.31***	6.91 ± 2.08	5.81 ± 0.37^#^	5.23 ± 2.21*
WBC (10^6^/mL)	8.94 ± 2.22	8.90 ± 1.03	8.62 ± 4.95	9.86 ± 3.11	14.4 ± 3.98	10.8 ± 4.22	8.53 ± 2.98	12.3 ± 1.55

The results are presented as means ± SD of three independent experiments and duplicate measurements.

*p < *0.05*, indicates that the mean is significantly different from the control by ANOVA with Kruskal–Wallis test followed by Dunn’s multiple comparisons test.

^*#*^*p* < *0.05*, indicates that the mean is significantly different from the LPS (25 μg/kg) group by ANOVA with Kruskal–Wallis test followed by Dunn’s multiple comparisons test.
